# Impact of delayed admission to intensive care units on mortality of critically ill patients: a cohort study

**DOI:** 10.1186/cc9975

**Published:** 2011-01-18

**Authors:** Lucienne TQ Cardoso, Cintia MC Grion, Tiemi Matsuo, Elza HT Anami, Ivanil AM Kauss, Ludmila Seko, Ana M Bonametti

**Affiliations:** 1 Hospital Universitário de Londrina, Divisão de Terapia Intensiva, Avenida Robert Koch 60, Vila Operária, Londrina, Paraná 86038-450, Brazil

## Abstract

**Introduction:**

When the number of patients who require intensive care is greater than the number of beds available, intensive care unit (ICU) entry flow is obstructed. This phenomenon has been associated with higher mortality rates in patients that are not admitted despite their need, and in patients that are admitted but are waiting for a bed. The purpose of this study is to evaluate if a delay in ICU admission affects mortality for critically ill patients.

**Methods:**

A prospective cohort of adult patients admitted to the ICU of our institution between January and December 2005 were analyzed. Patients for whom a bed was available were immediately admitted; when no bed was available, patients waited for ICU admission. ICU admission was classified as either delayed or immediate. Confounding variables examined were: age, sex, originating hospital ward, ICU diagnosis, co-morbidity, Acute Physiology and Chronic Health Evaluation (APACHE) II score, therapeutic intervention, and Sequential Organ Failure Assessment (SOFA) score. All patients were followed until hospital discharge.

**Results:**

A total of 401 patients were evaluated; 125 (31.2%) patients were immediately admitted and 276 (68.8%) patients had delayed admission. There was a significant increase in ICU mortality rates with a delay in ICU admission (*P *= 0.002). The fraction of mortality risk attributable to ICU delay was 30% (95% confidence interval (CI): 11.2% to 44.8%). Each hour of waiting was independently associated with a 1.5% increased risk of ICU death (hazard ratio (HR): 1.015; 95% CI 1.006 to 1.023; *P *= 0.001).

**Conclusions:**

There is a significant association between time to admission and survival rates. Early admission to the ICU is more likely to produce positive outcomes.

## Introduction

When the number of patients requiring intensive care management is greater than the number of beds available, ICU entry flow is obstructed [1] and the critically ill patient has to be cared for in hospital wards with non-specialized staff. Critically ill patients need early interventions to improve outcomes [2-7]; therefore, the phenomenon of waiting for ICU bed availability has been suggested to be associated with higher mortality [8-12]. The positive impact of ICU admission on patient survival is more evident during the first 72 hours of critical illness [13]. In the face of an aging and increasingly morbid global population [14], timely access to ICU beds becomes increasingly important [15,16].

The waiting time for ICU bed availability varies between hospitals and countries, and typically ranges from 2 hours to 3.5 days [8-12,17-19]. The proportion of patients who wait for ICU admission varies from 2.1 to 75.5% [8-12,20-22], depending on how delays are calculated. Some studies show no clear association between delayed admission and poor outcome [11,23]. Other studies report a five times higher risk of death, and a two times longer stay among patients not immediately admitted to the ICU [10].

It has been shown that patients meeting ICU admission criteria and treated in the ICU, compared to those treated out of the ICU, had a survival benefit [13]. There are few reports about delay in ICU admission due to obstruction of entry flow, especially in Latin American ICUs. Indeed, this public health care issue is becoming more prevalent in both developed [9,11,18,21] and developing countries [8,12].

The challenge of this study was to provide outcome data about critically ill patients who were initially treated in regular wards before an ICU bed became available. The aim of this study is to compare mortality rates of patients immediately admitted to the ICU with those who were required to wait for ICU bed availability.

## Materials and methods

This study was approved by the Londrina University Hospital Ethics Committee, which waived the requirement for informed consent.

### Setting and study design

We present a prospective cohort study of patients admitted to our 17-bed general adult ICU. The ICU staff consisted of certified intensivists who remained constant throughout the study. All patients were referred from our hospital; patients from other hospitals were not included.

### Inclusion and exclusion criteria

All patients consecutively admitted to the ICU from January to December 2005 were prospectively considered for inclusion in the study. Inclusion criteria for ICU admission were adopted from SCCM guidelines [24]. Exclusion criteria were: age less than 18 years of age; readmission to the ICU during the same hospitalization; patients who were transferred to other hospitals, who were considered to be lost to follow-up; elective surgery with prior assured access to the ICU (this group of patients has a lower risk of death [25] and would be allocated in the immediately admitted group, biasing interpretation of data); patients with less than 24 hours between ICU admission and discharge (death or less acuity); delay to admission longer than 72 hours, exceeding the suggested critical window of benefit [13,26].

### Data collection and definitions

Patients were immediately admitted if there was an ICU bed available. If not, the screening intensivist registered the request in an ICU access protocol and treatment was provided by the ward staff; ICU consultation in these cases was routinely part of the treatment. After ICU admission, patients were treated according to ICU protocols and all interventions were prospectively documented.

The need to wait for ICU admission due to bed unavailability was considered an exposure, and defined as the "delayed admission group". Those who were immediately admitted, or non-exposed, were defined as the "immediate admission group". Date and hour of the determination of ICU requirement were recorded, as well as that of ICU admission.

Patients who were required to wait for an ICU bed were admitted in chronological order, or on a "first come, first served" basis. This criterion was adopted based on the recommendations of the American Thoracic Society Bioethics Task Force. This recommendation specifically states that when the need for ICU beds exceeds available resources, patients should be admitted by arrival order [27]. Rearrangement of this order was allowed due to administrative or medical orders. For the immediate admission group waiting time was considered zero. The following demographic data were collected: sex, age, previous hospital length of stay, length of ICU stay, Acute Physiology and Chronic Health Evaluation (APACHE) II score and comorbidities [25], need for mechanical ventilation and tracheal intubation, vasoactive drug use, Therapeutic Intervention Scoring System (TISS) 28 score [28] on the first (TISS 28 D1) and last day of ICU, Sequential Organ Failure Assessment (SOFA) score [29] on the first day of ICU (SOFA D1). The hospital ward was stratified in two main categories: the emergency ward, composed of adult hospital beds for short hospital stays in the emergency department and general hospital wards.

The delayed admission group had two calculated APACHE II scores: the first score refers to the first 24 hours after ICU orders, and the second score used data collected during the first 24 hours after ICU admission. Follow-up continued until ICU, hospital discharge, and mortality rate was registered.

To independently evaluate age and comorbidities in multivariate analysis, the APACHE II score was dissociated with age, comorbidity, and Acute Physiology Score (APS) [30]. This approach was applied to the score calculated at the time of ICU ordering and at ICU admission.

Delay to ICU admission was also considered a continuous predictive variable in the Cox model of proportional risks. The primary outcome examined was ICU mortality. Other outcomes examined were hospital mortality, duration of mechanical ventilation, and length of stay in the ICU and hospital.

### Statistical analysis

Calculations of variables for cohort studies were performed with the Epitable program, (EpiInfo, version 6.04b, CDC, Atlanta, Georgia, USA) [31]. A total of 239 patients was calculated to detect a 20% reduction of absolute risk [11] with 95% confidence interval, 80% power, and a 1:2 non-exposure/exposure ratio.

Patient characteristics in the delayed and immediate admission groups were compared using non-paired t tests for continuous variables with normal distribution, the Mann-Whitney test for variables with non-Gaussian distribution, and the Wilcoxon rank sum test for paired samples of ICU ordering and admission scores in the delayed admission group. A normal distribution of variables was evaluated by the D'Agostino-Pearson test. Pearson's chi-square test was applied to categorical variables. The chi-square trend test was applied to analyze ICU mortality rate, according to delay categories. Association strength between delayed admission and mortality was described by relative risk. Impact of this association was described as attributable risk, according to the following formula: AR% = ((RR - 1)/RR) × 100 [31]. Multivariate Cox regression model was applied to evaluate delay to ICU admission and mortality considering confounding factors. A stepwise forward method was applied by entering relevant variables sequentially and after checking them, removing non-significant variables. A *P*-value of 0.05 was considered statistically significant. Data were entered on Epi Info (version 3.3.2, 2005, CDC, USA) and statistical analysis was performed on MedCalc for Windows (version 9.3.2.0, MedCalc Software, Mariakerke, Belgium) and SAS (version 8.2, SAS Institute, Cary, NC, USA).

## Results

**Figure 1:**
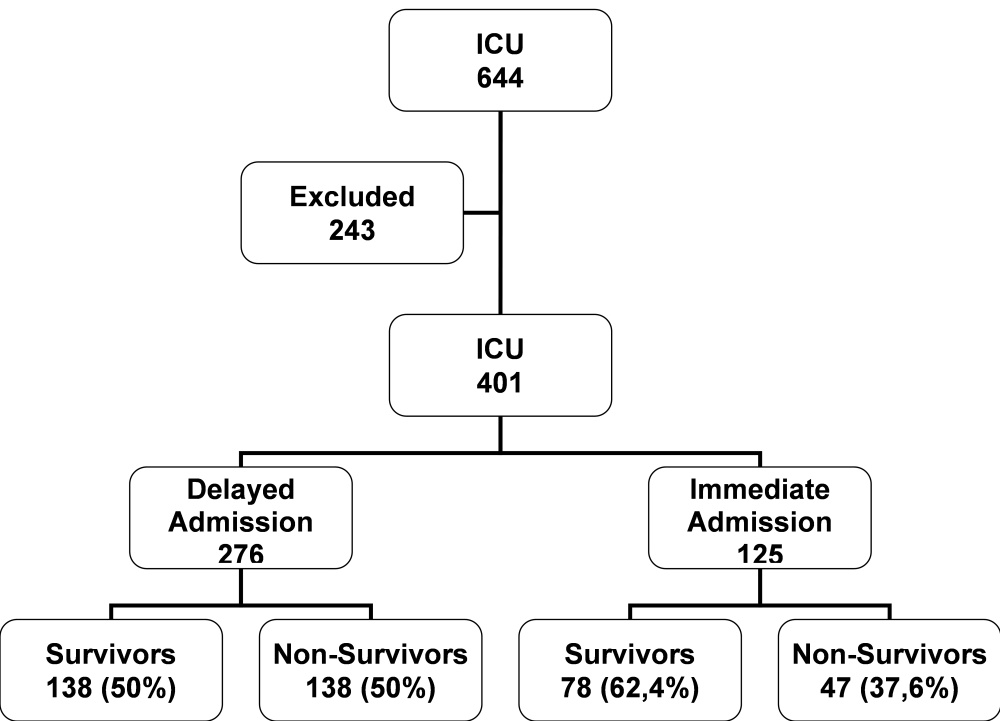
**Flow diagram of patient admissions**.

During the study period there were 644 ICU admissions. A total of 243 patients were excluded due to: 85 elective surgeries, 14 age less than18 years, 63 readmissions, 22 patients with a delay greater than 72 hours, 53 stayed less than 24 hours between ICU requirement and discharge, and 6 were lost to follow-up (Figure 1).

Mean occupation rate of ICU beds during the study period was 97.3%. The mean number of ICU admission orders per month was 58.4. The frequency of delayed admissions was 276/401 (68.8%). Duration of delay to ICU admission varied from 2.3 to 67.2 hours with a median delay of 17.8 hours (IQR, 7.6 to 31.2). Patients in the delayed admission group received medical care provided by ward staff while waiting for an available ICU bed. Essential procedures and investigations were performed: 62.3% mechanical ventilation, 55.1% vasoactive drugs and hemodynamic monitoring, 8% enteral nutrition, 1.5% dialysis, 67.3% antibiotics. Intracranial pressure monitoring, pulmonary artery catheters and intra-aortic balloon pumps were not available outside of the ICU.

General comparisons between patient groups are illustrated in Table 1. The length of hospital stay before ICU admission and comorbidities were both significantly higher in the delayed admission group (*P *= 0.002, *P *< 0.001, respectively). There was no significant difference in median duration of mechanical ventilation between patient groups (immediate = 6.0, IQR = 3 to 14 days; delayed = 6.5, IQR = 3 to 12; *P *= 0.565). Likewise, there was no significant difference in length of stay in either the ICU or the hospital (Table 1).

**Table 1:** Study sample characteristics at ICU admission

Patient characteristics	Delayed admission (*n *= 276)		Immediate admission (*n *= 125)		*P-*value
Male sex (n and %)	153	55.4	77	61.6	0.295
Age (years) (median and IQR)	61	42 to 72	60	43 to 73	0.913
Emergency department^a ^(n and %)	176	63.8	90	72.0	0.133
Length of hospital stay before ICU admission (days) (median and IQR)	2	1-6	0	0-1	0.002
Mechanical ventilation on first ICU day (n and %)	172	62.3	78	62.4	0.924
Mechanical ventilation before ICU (n and %)	155	56.2	69	55.2	0.944
Vasoactive drug use at first ICU day (n and %)	151	54.7	60	48.4	0.242
Co-morbidities (n and %)	70	25.4	13	10.4	<0.001
TISS 28 D1 (median and IQR)	22	17 to 27	22	17 to 26	0.977
TISS 28 at discharge^b ^(median and IQR)	15	13 to 17	15	13 to 17	0.390
APACHE II (median and IQR)	26	16.5 to 33	25	16 to 31	0.452
ICU length of stay (median and IQR)	5.0	2.0 to 10.5	4.0	2.0 to 10.0	0.519
Hospital length of stay (median and IQR)^c^	14.0	8.0 to 28.0	16.0	7.0 to 31.0	0.803

^a ^Emergency department room and emergency department ward.

^b ^ICU survivors.

^c ^Total hospital length of stay.

IQR, interquartile range; TISS 28 D1, TISS 28 in the first day of ICU stay.

Diagnoses were similar in both groups (Table 2). Although sepsis was the most frequent diagnosis in each group, it was more frequent in the delayed admission group (*P *= 0.005).

**Table 2:** Distribution of most frequent diagnosis according to APACHE II score among delayed and immediate admission groups

**Diagnostic category**^**a**^	Delayed admission		Immediate admission		*P*-value
			
	N	%	N	%	
MVOS^b ^- Cardiovascular	4	1.40%	5	4.00%	0.213
Diabetic ketoacidosis	4	1.40%	0	0.00%	0.421
MVOS^b ^- Gastrointestinal	1	0.40%	3	2.40%	0.171
Intracranial hemorrhage	18	6.50%	6	4.80%	0.669
Congestive heart failure	5	1.80%	0	0.00%	0.307
Coronary artery disease	21	7.60%	11	8.90%	0.817
MVOS^b ^- Neurologic	19	6.90%	14	11.30%	0.199
Multiple trauma	3	1.10%	3	2.40%	0.569
Postcardiac arrest	8	2.90%	5	4.00%	0.774
Gastrointestinal bleeding	2	0.70%	3	2.40%	0.355
Sepsis	172	62.30%	58	46.80%	0.005
Head trauma	6	2.20%	5	4.00%	0.471

^a ^Diagnostic categories of APACHE II system as originally described by Knaus *et al.*

^b^MVOS, Major vital organ system.

There was a significant increase in SOFA and APACHE II scores between the time of ICU ordering and admission (Supplementary Table in Additional file 1). However, these scores did not differ in the first day of ICU between immediate and delayed admission groups.

**Figure 2:**
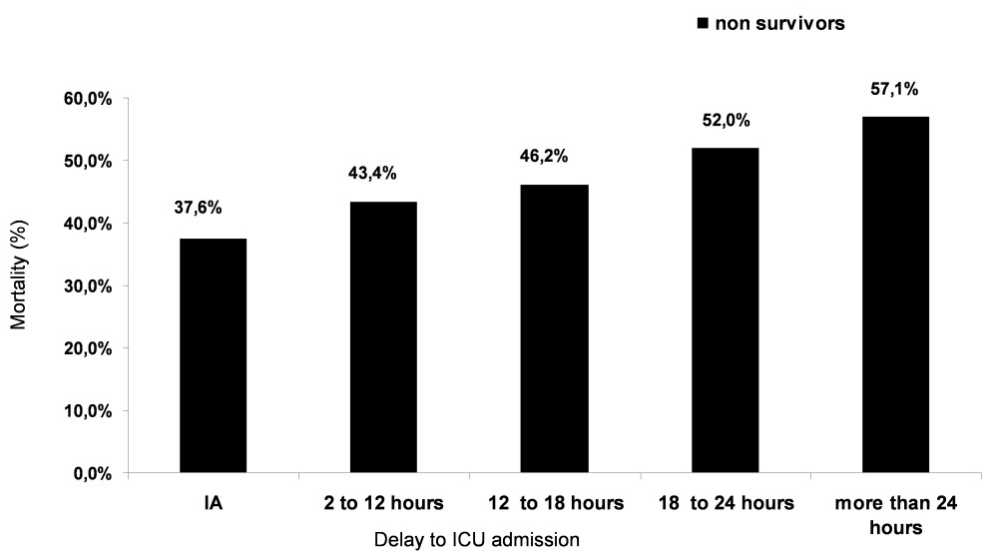
**ICU mortality rate among patients grouped by time to ICU admission**. This figure shows increase in mortality rate according to ICU waiting time. There is a significant tendency of increase in mortality with time. IA, immediate admission (c2: 9.78; *P *= 0.002).

ICU mortality rates increased with delay for ICU admission intervals (*P *= 0.002) (Figure 2). Bivariate analysis showed that the attributable fraction for ICU mortality risk, adjusted for the severity of illness, was 30.0% (CI 95%: 11.2 to 44.8%).

Analysis of the delay to ICU admission by multivariate analysis is presented in Table 3. Each waiting hour was associated independently with a 1.5% increase in risk of ICU mortality (hazard ratio = 1.015; 95% CI: 1.006 to 1.023; *P *= 0.001). Another variable independently associated with survival rate was SOFA score.

**Table 3:** Univariate and multivariate analysis by Cox Regression Model of ICU mortality risk factors

	Univariate			Multivariate		
		
Variables	HR	(95% CI)	*P*-value	HR-	(95% CI)	*P*-value
				**adjusted**^***a***^		
Waiting time	1.013	1.005 to 1.022	0.003	1.015	1.006 to 1.023	0.001
Male sex	1.068	0.796 to 1.433	0.663			
Age (years)	1.006	0.998 to 1.014	0.133			
Comorbidities	1.585	1.128 to 2.229	0.008			
APS score	1.043	1.026 to 1.060	<0.001			
SOFA score	1.103	1.064 to 1.143	<0.001	1.103	1.065 to 1.143	<0.001
TISS 28 score	1.051	1.030 to 1.073	<0.001			
General hospital ward^b^	1.311	0.979 to 1.756	0.071			
Length of hospital stay before ICU (days)	1.005	0.989 to 1.021	0.524			
Sepsis diagnosis	1.493	1.073 to 2.077	0.018			

χ^2 ^= 38.7512, 2 g. l., *P-*value < 0.001.

^*a *^adjusted to waiting time (hours), age (years), co-morbidities, severity of illness, organ dysfunction, therapeutic interventions, hospital ward origin, hospital length of stay before ICU, and sepsis diagnosis.

^b ^Hospital ward origin, outside emergency department.

ICU, Intensive Care Unit; HR, hazard ratio; APS, Acute Physiology Score; SOFA, Sequential Organ Failure Assessment; TISS 28, Therapeutic Intervention Scoring System.

A similar association was found when applying multivariate analysis to evaluate risk factors to hospital mortality; each hour of delay was independently associated with a 1.0% increase in risk of hospital death (hazard ratio = 1.010; 95% CI: 1.002 to 1.018; *P *= 0.014). In this model, additional variables independently associated with mortality were age, SOFA score, and general hospital ward.

## Discussion

In our study, delay of ICU admission due to unavailability of ICU beds is a common occurrence. There is an association between delay to ICU admission and higher mortality rate.

Effective access to health care systems is comprised of three components, which must be equally adequate: care, timing, and location [15,16]. In our study we assumed that health care access was not adequate due to the timing of ICU admission. Our data emphasize the importance of providing early, specialized intervention to prevent organ dysfunction and to reduce risk factors leading to mortality. Despite the care provided by ward staff while patients were waiting for ICU bed availability, these healthcare providers were not trained in critical care and were not as experienced in caring for ICU patients. Patients in the delayed admission group experienced an increase in SOFA score while waiting, reflecting worsening of organ dysfunction during this period.

General hospital wards are neither designed nor staffed to provide extended longitudinal care for the critically ill patient [9]. These patients have better outcomes when treated in ICUs with close and continuous involvement by critical care physicians [32,33]. Other data also show improved outcome when nurse-to-patient ratios in the ICUs are properly maintained [34].

Caring for critically ill patients outside the ICU may also imply an increased burden and high stress level experienced by hospital ward staff. Furthermore, patients admitted and treated outside the ICU are reimbursed as regular admissions by our health care system; costs are predictably higher when patients become critical. This budget deficit must be covered by hospital managers, generating financial difficulties.

Most studies of ICU triage have focused on patients admitted [11,30,35] or rejected for ICU management [13,36], which prevents comparison with patients who have been transferred late to the ICU. Our study evaluated the impact of delay to ICU admission on mortality, when patients are admitted at a later point, pending bed availability. We demonstrated an increase in mortality by each hour of waiting time.

Even in countries such as the United States, where there is no shortage of ICU beds, it has been reported that a more than six-hour delay in intensive care unit transfer increased hospital length of stay and ICU and hospital mortality [9]. Young *et al*. [10] found a 3.5 higher non-adjusted mortality in patients with four or more hours of delay to treatment after physiological deterioration. There was one major difference between our data and these studies, as we did not find an increase in length of ICU or hospital stay in the delayed admission group. This may be the result of interventions started already at the ward while the patients were waiting for the ICU bed.

Engoren [35] also did not detect differences in length of ICU or hospital stay between patients who were evaluated within six hours, and those that waited more than six hours before physician evaluation. Similar to our study, patients were already receiving specialized care, although there was a delay to intensivist evaluation, which resulted in a 1.6% higher risk of death per hour of waiting.

The frequency of delay to ICU admission is considered high in our study when compared with data reported from several other countries. Previously reported incidence rates in Israel (24 to 56.5%) [11,20], France (37.6%) [37], England (32.6%) [21], and Hong Kong (37.8%) [22] are all lower than that of our Brazilian study (68.8%). Interestingly, our results are consistent with previous work from Brazil [8] in a cohort of patients submitted to emergency surgery (75.5%).

The 68.8% frequency of delayed admission reflects the 97.3% occupation rate of ICU beds [38] in our institution, which is above the 80% recommended by the World Health Organization [39]. This high occupation rate means there is rarely a bed available for immediate admission. Our patient characteristics are similar to those of other studies; and we have higher mean severity of illness scores compared to other studies [8-10,12].

Our country has a nationalized health care system so that every citizen should have equal access. Intensive care treatment consumes a large part of our health care resources, so it must be used equitably. We demonstrate that late admission of critically ill patients to an ICU results in increased mortality. Another important consideration is that the number of ICU beds required is often based on theoretical calculations rather than actual patient data [40]. A British study estimated a two-fold increase in the number of ICU beds required for a region [41] and we speculate that our institution requires a similar increase since delay due to unavailability of ICU beds was very high.

There are several limitations to our study. First, we analyzed data from a single center, so there is low external validity. However, our results are consistent with other publications. Second, observational studies are susceptible to selection bias, which can interfere with results. Indeed, the access protocol constituted a waiting list organized in chronological order, which should result in similar characteristics for both groups, except for the presence of sepsis and comorbidities that were more frequently found in the delayed admission group. Despite these differences, APACHE II scores and probabilities of death were similar in both groups at the time of study entry. Third, our designation of delay in the immediate admission group as zero may have caused an underestimation of the association between waiting time and mortality. This occurred because the zero designation was actually a lack of measurement of real time to admission when an ICU bed was available. The most obvious limitation of this study is the small numbers of critically ill patients included, which make careful interpretation necessary.

## Conclusions

Delay in ICU admission or intensive care due to unavailability of beds is common in our institution. The present study shows an independent association between delayed admission and higher mortality, even if the patient is eventually admitted to the ICU. Each hour of delay is associated with an increase in mortality. Early access to intensive care greatly benefits critically ill patients.

## Key messages

• Demands for ICU beds are increasing worldwide and delay to ICU admission is becoming a more frequent issue.

• There is an increase in mortality for each hour of delay to ICU access.

• Critically ill patients show further physiologic deterioration and an increase in organ dysfunction while waiting for an ICU bed to become available.

## Abbreviations

APACHE II: Acute Physiology and Chronic Health Evaluation; APS: Acute Physiology Score; AR: attributable risk; CDC: Centers for Disease Control and Prevention; CI: confidence interval; HR: hazard ratio; ICU: intensive care unit; RR: relative risk; SAS: Statistical Analysis System; SCCM: Society of Critical Care Medicine; SOFA D1: Sequential Organ Failure Assessment in the first day of ICU stay; SOFA: Sequential Organ Failure Assessment; TISS 28 D1: Therapeutic Intervention Scoring System 28 in the first day of ICU stay; TISS 28: Therapeutic Intervention Scoring System 28.

## Competing interests

The authors declare that they have no competing interests.

## Authors' contributions

LTQC, TM and AMB participated in the study concept and design. CMCG, LS, EHTA, and IAMK carried out the acquisition of data and participated in the analysis and interpretation of data. LTQC and CMCG drafted the manuscript. LTQC and TM performed the statistical analysis. All authors participated in critical revision of the manuscript for intellectual content, and approved the final version of the manuscript.

## Supplementary Material

Additional file 1 **Analysis of APACHE II and SOFA Score at ICU Ordering and Admission**. Supplementary Table comparing APACHE II and SOFA scores at the time of ICU ordering and on ICU admission between the two groups of patients (delayed and immediate admission). Click here for file
