# Supplementary material for: Impact of delayed admission to intensive care units on mortality of critically ill patients: a cohort study
Source: Crit Care. 2011 Jan 18;15(1):R28. doi: 10.1186/cc9975 (PMC3222064; doi:10.1186/cc9975)
Supplement: Additional file 1 — Analysis of APACHE II and SOFA Score at ICU Ordering and Admission. Supplementary Table comparing APACHE II and SOFA scores at the time of ICU ordering and on ICU admission between the two groups of patients (delayed and immediate admission). [file cc9975-S1.DOC]

Supplementary Table – Analysis of APACHE II and SOFA Score at ICU Ordering and Admission

|  | **ICU ordering** | | | | | **ICU admission** | | | | |  |
| --- | --- | --- | --- | --- | --- | --- | --- | --- | --- | --- | --- |
|  | **Delayed Admission**  **(n = 276)** | | **Immediate Admission**  **(n = 125)** | | **p**^a^ **value** | **Delayed Admission**  **(n = 276)** | | **Immediate Admission**  **(n = 125)** | | **p**^a^ **value** | **p**^b^ **value** |
| **SOFA D1**  (median and IQR) | 7 | 3.5 - 11 | 8 | 4 - 11 | 0.234 | 9 | 5 - 12 | 8 | 4 - 11 | 0.228 | < 0.001 |
| **APACHE II** (median and IQR) | 23 | 14.5 - 32 | 25 | 16 - 31 | 0.575 | 26 | 16.5 - 33 | 25 | 16 - 31 | 0.452 | 0.049 |
| **Death risk^c^** (median and IQR) | 0.404 | 0.182 - 0.780 | 0.5110 | 0.229 - 0.748 | 0.815 | 0.562 | 0.234 - 0.804 | 0.501 | 0.229 - 0.748 | 0.380 | 0.001 |

^a^ Mann-Whitney test, comparing Delayed and Immediate Admission

^b^ Wilcoxon’s test for ICU ordering and admission scores, among delayed admission group.

^c^ Death risk calculated with APACHE II score [29]

IQR – interquartile range
